# Hepatitis C treatment: where are we now?

**DOI:** 10.2147/IJGM.S127689

**Published:** 2017-02-17

**Authors:** Nicholas J Burstow, Zameer Mohamed, Asmaa I Gomaa, Mark W Sonderup, Nicola A Cook, Imam Waked, C Wendy Spearman, Simon D Taylor-Robinson

**Affiliations:** 1 Liver Unit, Department of Surgery and Cancer, Imperial College London, London, UK; 2 National Liver Institute, Menoufiya University, Shbeen El Kom, Egypt; 3 Division of Hepatology, Department of Medicine, Faculty of Health Sciences, University of Cape Town and Groote Schuur Hospital, Cape Town, Republic of South Africa

**Keywords:** hepatitis C, protease inhibitors, directly acting antivirals, interferon-free regimens, ribavirin-free regimens, hepatitis C eradication

## Abstract

Chronic hepatitis C infection affects millions of people worldwide and confers significant morbidity and mortality. Effective treatment is needed to prevent disease progression and associated complications. Previous treatment options were limited to interferon and ribavirin (RBV) regimens, which gave low cure rates and were associated with unpleasant side effects. The era of direct-acting antiviral (DAA) therapies began with the development of first-generation NS3/4A protease inhibitors in 2011. They vastly improved outcomes for patients, particularly those with genotype 1 infection, the most prevalent genotype globally. Since then, a multitude of DAAs have been licensed for use, and outcomes for patients have improved further, with fewer side effects and cure rates approaching 100%. Recent regimens are interferon-free, and in many cases, RBV-free, and involve a combination of DAA agents. This review summarizes the treatment options currently available and discusses potential barriers that may delay the global eradication of hepatitis C.

## Introduction

Chronic infection with hepatitis C virus (HCV) is estimated to affect up to 177.5 million people worldwide.^1^ While a small proportion of people clear the virus naturally, chronic hepatitis C (CHC) can lead to a spectrum of liver diseases from mild inflammation with a relatively indolent course to extensive liver fibrosis and consequent cirrhosis, conferring significant morbidity and mortality to affected individuals. With end-stage liver disease, the manifestations of hepatic decompensation are common. Associated hepatocellular carcinoma is a serious complication of CHC-related cirrhosis with an incidence of 5.8% per year in the at-risk population.^2^ Such disease progression is particularly problematic for CHC patients, as the infection is often asymptomatic and only diagnosed when the pathological processes are relatively advanced.

**Figure 1:**
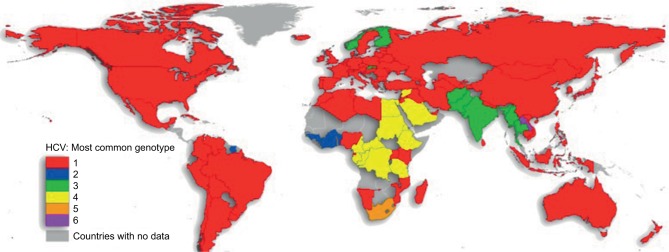
Genotype 1 is the most common cause of chronic hepatitis C infection worldwide. Reproduced from Messina JP, Humphreys I, Flaxman A, et al. Global distribution and prevalence of hepatitis C virus genotypes. *Hepatology*. 2015;61(1):77–87. Creative Commons license and disclaimer available from: http://creativecommons.org/licenses/by/4.0/legalcode.^5^ **Abbreviation:** HCV, hepatitis C virus.

There are six major, structurally different and clinically relevant HCV genotypes, with several subtypes being described.^3^ In addition, recently, four genotype (GT) 7 patients have been reported in the Democratic Republic of Congo.^4^ GT1 accounts for the majority of cases worldwide (Figure 1).^5^ Distinction between genotypes remains important because treatment regimens are mostly still genotype specific.

Interferon-based regimens, and later with the addition of ribavirin (RBV), were the standard CHC treatment for many years. However, treatment outcomes varied greatly between genotypes, with particularly poor cure rates of 40% being reported in GT1 and GT4 cases.^6^,^7^ Since 2011, a number of directly acting antivirals (DAAs) have been licensed for use as part of combination therapies for CHC, and outcomes for patients have improved considerably.

## Global distribution of hepatitis C genotypes

**Figure 2:**
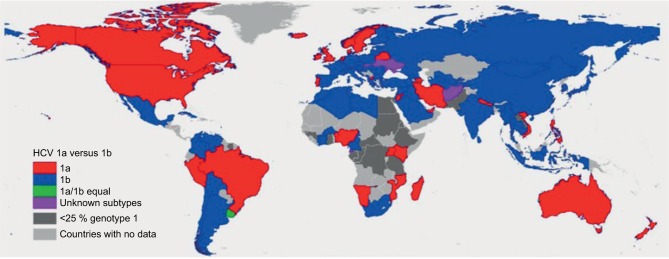
Distribution of GT1a versus GT1b. Reproduced from Messina JP, Humphreys I, Flaxman A, et al. Global distribution and prevalence of hepatitis C virus genotypes. *Hepatology*. 2015;61(1):77–87. Creative Commons license and disclaimer available from: http://creativecommons.org/licenses/by/4.0/legalcode.^5^ **Abbreviations:** GT, genotype; HCV, hepatitis C virus.

HCV follows a worldwide distribution, with Africa and Central and East Asia being most affected.^8^ The most common genotype both worldwide and in Europe and North America is GT1, accounting for 49.1% of CHC cases.^1^ GT1 infection can be further subdivided into two major classes: 1a and 1b.^3^ While GT1a accounts for the majority of CHC GT1 cases in North America, the majority of CHC GT1 cases worldwide are due to GT1b (68% versus 31% GT1a)^5^ (Figure 2). GT3 is the second most common genotype globally, accounting for 17.9% of CHC cases. Worldwide, GT4, GT2, and GT5 account for 16.8%, 11%, and 2% of cases, respectively.^1^ According to recent estimates, GT6 infection is the least common, accounting for 1.4% of CHC cases.^1^ Genotype distributions in Europe follow a similar pattern, with GT1 and GT3 accounting for the majority of CHC cases (64.4% and 25.5%, respectively).^9^ Globally, the majority of GT2 and GT6 cases are found in East Asia. GT4 is most commonly found in North Africa and the Middle East, particularly in Egypt following the anti-schistosomal treatment program that left many millions infected with HCV.^5^,^10^ GT5 is primarily found in South Africa.^5^

## Modes of HCV transmission

Health care-associated transmission, through unsterilized needles or transfusion with contaminated blood, remains a major route of HCV infection, particularly in low- and middle-income countries (LMICs).^10^–^12^ While uncommon in high-income settings, iatrogenic infection has also been reported in European countries including France and Italy,^13^,^14^ and in isolated hospital outbreaks in the US.^15^,^16^ Indeed, a study of CHC patients in southern Italy showed surgery and dental therapy to be important risk factors for HCV infection.^17^ People who inject drugs, carrying out high-risk activities such as needle sharing, also account for a significant number of worldwide infections. Principally, this has been the most important factor in the developed world.^18^ However, more recently, emerging intravenous drug usage in LMICs has been shown as an important vector for HCV transmission.^19^ Other modes of HCV transmission include vertical mother-to-infant transmission, men who have sex with men, and the increasingly common trend of body art with tattooing.^12^

## HCV structure

**Figure 3:**
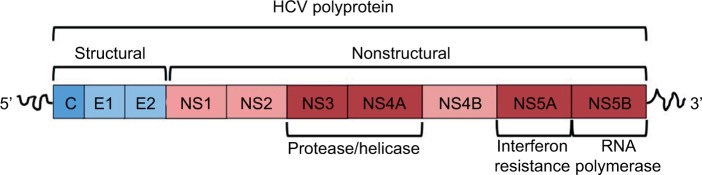
Hepatitis C virus polyprotein structure. **Abbreviation:** HCV, hepatitis C virus.

Understanding the structure of HCV is particularly important because newer therapies target specific viral proteins. HCV is an enveloped, single-stranded RNA virus. Its positively stranded genome encodes a polyprotein, comprising roughly 3,000 amino acids.^20^ This polyprotein is posttranslationally modified by proteolytic enzymes into four structural and six nonstructural (NS) proteins (Figure 3).^21^ Of particular importance are the NS3/4A, NS5A, and NS5B proteins. The NS3/4A serine protease mediates cleavage of the 3,000-amino-acid polyprotein into its respective structural and NS proteins. NS5B is responsible for RNA-dependent RNA polymerase activity, while NS5A is thought to have a number of roles including mediating interferon resistance.^21^ Ultimately, these viral proteins work together to drive viral replication and persistence.

## Diagnosis of chronic HCV infection

HCV infection may follow an acute or chronic course. Chronic HCV infection is diagnosed based on the presence of anti-HCV antibodies as a screening procedure and quantitative HCV RNA as the definitive test. After 6 months, anti-HCV antibodies are detectable through enzyme immunoassay. HCV RNA is detected using a sensitive molecular method such as polymerase chain reaction (PCR), with a lower limit of detection of <15 IU/mL.^22^

## The need for treatment

Given the increased morbidity and mortality associated with CHC infection, achieving viral clearance is critical and is associated with significantly reduced rates of liver failure and liver-related deaths amongst CHC patients.^23^ Viral clearance also significantly reduces fibrosis progression rates and even reverses cirrhosis.^24^ This explains why risks of all-cause mortality amongst patients with cirrhosis have also been shown to be lower in those successfully clearing the HCV infection.^25^ Virological clearance of infection with undetectable HCV RNA levels 3–6 months after completing antiviral treatment is termed a sustained virological response (SVR), and this defines a cure.^26^,^27^ The concordance of SVR12 (at 12 weeks following end of treatment) and SVR24 (at 24 weeks following end of treatment) as end points of CHC therapy is 99%, meaning that both are acceptable markers of viral clearance.^27^

## Treatment options

### Initial treatment options: interferon and RBV

Until the early 1990s, there was no treatment available for CHC. It was during this decade that the benefits of interferon-alfa therapy were reported, leading to a recommended treatment regimen, comprising a 24- or 48-week course of interferon-alfa 2a or 2b, depending on genotype.^28^ Patients required three times weekly injections, and outcomes were poor, with ≤10% of patients successfully clearing the virus.^29^ The addition of RBV to interferon-alfa therapy considerably improved outcomes, increasing SVR rates to approximately 30–40%.^7^

#### Pegylated interferon and RBV

The development of pegylated interferon (PEG-IFN)-alfa 2a and 2b toward the end of the 1990s altered the kinetics of interferon, meaning that patients only required a single weekly injection.^30^ It also improved clearance rates and, up until 2011, a 24- or 48-week course of PEG-IFN-alfa 2a or 2b plus RBV was the standard of care for CHC infection.^31^ SVR rates of up to 80% were reported for GT2, GT3, GT5, and GT6, with GT2 having the highest cure rates.^32^,^33^ Intermediate rates of SVR were reported for GT4.^33^ However, patients with CHC GT1, the most common genotype worldwide, achieved much lower rates of SVR of around 40%.^6^,^7^ Rates of SVR were even lower amongst Afro-Caribbean CHC GT1 patients.^34^ In addition to the variable SVR rates,^6^,^7^,^32^,^33^ several contraindications meant that PEG-IFN plus RBV therapy was not suitable for a number of patients. Owing to the potential neuropsychiatric effects of interferon, PEG-IFN plus RBV therapy is contraindicated in patients with uncontrolled depression or psychosis. Given the immune-modulatory effect of interferon, therapy is contraindicated in patients with autoimmune disease. In individuals with cirrhosis, interferon can precipitate decompensation due to increased necro-inflammation and hepatocyte necrosis. Interferon therapy is thus contraindicated in patients with decompensated liver disease.^28^ A complete list of contraindications can be found in the European Association for the Study of the Liver (EASL) Recommendations on Treatment of Hepatitis C 2015 guidelines.^32^

### DAAs: first-generation, “first-wave” protease inhibitors

The poor outcomes reported amongst CHC GT1 patients, who account for the majority of CHC patients worldwide,^6^,^7^ drove the need for newer, more effective treatments for CHC infection. The first such drugs to be developed were the protease inhibitors (PIs) boceprevir (BOC) and telaprevir (TVR). These were first-generation, first-wave DAAs, licensed to treat CHC GT1 infection in 2011.^32^ Both BOC and TVR are NS3/4A inhibitors. The NS3/4A serine protease plays a key role in HCV replication, cleaving the viral polyprotein into its constituent parts.^21^ The resultant structural and NS proteins are responsible for viral replication and persistence. By targeting and inhibiting the NS3/4A protease, BOC and TVR are able to mediate viral clearance.^20^,^35^ In order to prevent the emergence of viral resistance,^36^,^37^ BOC and TVR are given alongside PEG-IFN plus RBV as part of “triple therapy”.

#### Boceprevir

The antiviral capacity of BOC was first demonstrated in replicon cell models^38^ and later confirmed in phase I and II clinical trials.^37^,^39^ The SPRINT-2 phase III trial investigated BOC-based triple therapy amongst CHC GT1 treatment-naïve patients.^40^ SVR24 rates of 63% (233/368) were reported, compared to 38% (137/363) in patients receiving traditional PEG-IFN plus RBV therapy.^40^ The RESPOND-2 phase III trial investigated outcomes of CHC GT1 treatment-experienced patients receiving BOC-based triple therapy.^41^ SVR24 rates of 66% of patients were reported in those receiving 48 weeks of therapy, versus 21% in the PEG-IFN plus RBV control group.^41^ While achieving higher rates of SVR, BOC therapy was associated with increased frequency of adverse events (AEs). In particular, anemia and dysgeusia were significantly more common in those receiving BOC.^40^,^41^

#### Telaprevir

Early phase I and II clinical trials demonstrated the antiviral capacity of TVR.^42^–^46^ The ADVANCE phase III trial investigated TVR-based triple therapy amongst CHC GT1 treatment-naïve patients.^47^ SVR24 was achieved in 75% (271/363) of TVR patients, versus 44% (158/361) of those receiving traditional therapy.^47^ The REALIZE phase III trial investigated treatment outcomes of CHC GT1 patients receiving TVR.^48^ SVR24 rates of 64% in TVR patients, compared to 17% in the control group, were reported.^48^ As was the case with the other first-wave PI BOC, AEs, especially rash and anemia, were more common in those receiving TVR.^40^,^41^,^47^,^48^

### The problem with first-generation, first-wave PIs – need for alternatives

The first-generation, first-wave NS3/4A PIs BOC and TVR improved outcomes for CHC GT1 patients. SVR rates increased from 40% with traditional interferon-based therapy^6^,^7^ to between 64% and 75% using triple therapy with BOC or TVR.^40^,^41^,^47^,^48^ However, these regimes were limited to CHC GT1, and were associated with frequent side effects including anemia, fatigue, and rash, with consequently high discontinuation rates.^40^,^41^,^47^,^48^ Dosing regimens were complex, and tablets had to be taken with fatty meals every 8 hours. These complex regimens also conferred drug–drug interactions, complicating coexisting treatments for other conditions. Poorer outcomes were reported in patients with advanced fibrosis and cirrhosis.^40^,^41^,^47^,^48^ Real-life data reported inferior outcomes, compared to those in phase III trials, with SVR being achieved in 40–53% and 53–56% of BOC and TVR patients, respectively.^49^,^50^ Serious AEs were more frequent in real-life treatment-experienced patients with cirrhosis receiving BOC/TVR triple therapy.^51^ Consequently, there was a clear need for more tolerable and effective CHC treatments.

### New components to antiviral therapy

Since the release of the first-wave, first-generation PIs BOC and TVR in 2011, there have been a number of DAAs licensed for the treatment of CHC (Table 1).

### NS3/4A inhibitors

#### Simeprevir

Sharing the same target as its predecessors, BOC and TVR, simeprevir (SMV) is a second-wave, first-generation NS3/4A PI.^52^ Initial phase I studies demonstrated its antiviral capacity in CHC GT1 patients.^53^ Subsequent phase II studies confirmed these findings, and also demonstrated antiviral activity in GT2 and GT4–6 patients.^54^,^55^

SMV may be given alongside PEG-IFN plus RBV as part of SMV-based triple therapy. This regime was investigated in the QUEST-1 and QUEST-2 phase III clinical trials.^56^,^57^ Participants were all treatment-naïve CHC GT1 patients. Eighty percent (210/264) and 81% (209/257) of patients achieved SVR, respectively.^56^,^57^ When data from both trials were pooled and analyzed, 85% (228/267) of GT1b patients found to have achieved SVR.^32^ Outcomes of GT1a patients varied depending on the status of the Q80K polymorphism. This is a naturally occurring polymorphism within the HCV NS3 protease domain, associated with reduced activity of NS3/4A PIs.^58^ Fifty-eight percent (49/84) of GT1a patients with the Q80K polymorphism and 84% (138/165) of cases without the polymorphism achieved SVR.^32^ SMV-based triple therapy has also been used to treat CHC GT4 patients. The RESTORE phase III trial investigated outcomes of a mixture of treatment-naïve and treatment-experienced CHC GT4 patients.^59^ Outcomes were favorable in treatment-naïve and prior relapsers, with 83% (29/35) and 86% (19/22) achieving SVR12, respectively. However, only 60% (6/10) of prior partial responders and 40% (16/40) of prior null responders achieved SVR12.^59^

#### Grazoprevir

Grazoprevir (GZR) is a second-generation NS3/4A PI that demonstrates antiviral activity against all major genotypes in vitro.^60^ GZR triple therapy with PEG-IFN plus RBV in CHC GT1 patients without cirrhosis was investigated in an early phase II study. SVR24 was achieved in 89–93% of patients, depending on the GZR dose received.^61^ The C-SPIRIT study investigated outcomes of CHC GT1 patients receiving the interferon-free, GZR plus RBV combination.^62^ Results were good in patients with undetectable HCV RNA 4 weeks into treatment, with 90% (9/10) achieving SVR. However, patients with detectable RNA at week 4 fared less well, with 58% (7/12) achieving SVR.^62^

#### Paritaprevir

The final NS3/4A PI licensed for CHC treatment is paritaprevir (PTV). There are limited data regarding PTV therapy with PEG-IFN plus RBV or RBV, as PTV is coadministered with other antiviral agents.

#### Asunaprevir, voxileprevir, and glecaprevir

Asunaprevir (ASN), voxileprevir (VOX), and glecaprevir (GLC) are some of the last remaining NS3/4A PIs in development. There are limited data regarding ASN/VOX/GLC therapy with PEG-IFN plus RBV or RBV, as these PIs are given alongside other DAAs. Phase III trial data show ASN in combination with other DAAs to be an effective treatment for patients with cirrhosis and GT1 infection.^63^ Phase II data has demonstrated promising outcomes for GT1 and GT3 patients treated with VOX- or GLC-based DAA regimes.^64^,^65^ We await the results of phase III trial data to confirm these findings.

### NS5A inhibitors

#### Daclatasvir

Daclatasvir (DCV) is a pangenotypic NS5A inhibitor. The HCV NS5A protease has a number of roles essential for viral replication, including mediating interferon-resistance, thereby precipitating viral persistence.^21^ Thus, targeting and inhibiting the NS5A protease offers a potential route of viral clearance. The antiviral capacity of DCV in GT1 patients was demonstrated in an early phase II study, where it was given alongside PEG-IFN plus RBV as part of DCV-based triple therapy.^66^ A subsequent phase II study confirmed the efficacy of DCV-based triple therapy in CHC GT1 patients, and also added to previous findings by demonstrating effectiveness amongst GT4 patients.^67^ Phase III clinical trials investigating DCV-based triple therapy are yet to be done, as recent research is focused on combining DCV with other DAAs.

#### Ledipasvir, elbasvir, ombitasvir, velpatasvir, and odalasvir

Ledipasvir (LDV), elbasvir (EBR), ombitasvir (OBV), velpatasvir (VEL), and odalasvir are all NS5A inhibitors with varying genotype activity, particularly against GT1 and GT4 infection. Demonstrating antiviral activity in early studies,^68^–^71^ these NS5A inhibitors are given in combination with a variety of other DAAs.

### NS5B inhibitors

#### Sofosbuvir

Sofosbuvir (SOF) is a pangenotypic nucleotide analog inhibitor of HCV NS5B viral polymerase. The HCV NS5B polymerase is an RNA-dependent RNA polymerase, which facilitates RNA synthesis during HCV replication.^72^ Therefore, inhibition of the HCV NS5B polymerase offers significant antiviral potential. As with other DAAs, SOF may be given alongside PEG-IFN plus RBV as part of SOF-based triple therapy. While SMV is only used for CHC GT1 and GT4 patients, SOF demonstrates pangenotypic antiviral activity in vitro^72^ which has been confirmed in subsequent clinical trials.^73^–^75^

The NEUTRINO phase III trial investigated outcomes of SOF-based triple therapy amongst treatment-naïve CHC GT1 and GT4–6 patients.^73^ Of the 327 patients included, 291 (89%) were infected with GT1 (225 GT1a and 66 GT1b), 28 (9%) with GT4, one with GT5 (1%), and six with GT6 (6%). SVR was achieved in 89% (259/291) of CHC GT1 patients. Within those infected with GT1 subtypes, SVR was achieved in 92% (207/225) and 82% (54/66) of GT1a and GT1b patients, respectively. Ninety-six percent (27/28) of GT4 patients achieved SVR. Both the single GT5 patient and six GT6 patients achieved SVR.^73^

SOF-based triple therapy has also been shown to be effective in CHC GT2 and GT3 patients. In a phase II study of 23 treatment-experienced GT2 patients, 96% achieved SVR.^75^ This study also investigated outcomes of GT3 patients, with 83% (20/24) patients achieving SVR.^75^ The effectiveness of SOF-based triple therapy in CHC GT3 was confirmed in a second phase II study, where nine of 10 treatment-naïve CHC GT3 patients achieved SVR.^74^ The remaining patient was lost to follow-up. Phase III trials investigating this regime have yet to be published at the time of writing.

SOF may also be given with RBV as part of an interferon-free regime. This combination has been used in the treatment of GT2–4 infection. Phase III trials involving GT2 patients reported SVR rates between 86% and 97% following a 12-week course of SOF plus RBV.^73^,^76^,^77^ These phase III trials also investigated outcomes of GT3 patients treated with SOF plus RBV. The FISSION and POSITRON trials reported SVR rates of 56% (102/183) and 61% (60/98), respectively, with a 12-week course of SOF plus RBV.^73^,^76^ The FUSION trial^76^ compared outcomes of 12-week versus 16-week SOF plus RBV therapy amongst GT3 patients, and found longer treatment duration to be associated with higher rates of SVR (30% versus 62%, respectively). As a result of these findings, the VALENCE trial increased treatment duration to 24 weeks, and reported SVR rates of 85% (213/250) amongst GT3 patients.^77^ Trials involving Egyptian patients with GT4 infection treated for 12 and 24 weeks with SOF plus RBV reported SVR rates of 68–77% and 90–93%, respectively.^78^,^79^

The combination of SOF plus RBV was well tolerated, with few patients stopping treatment due to side effects. GT2 and GT4 patients achieved high rates of SVR, and while rates of SVR were lower amongst GT3 patients, outcomes were improved with longer treatment durations.^73^,^76^–^79^

#### Dasabuvir

Dasabuvir (DVR) is a non-nucleoside NS5B polymerase inhibitor.^80^ This DAA is given in combination with other DAAs to mediate viral clearance, and has been shown to be particularly effective in treating CHC GT1.^81^–^83^

### The dawn of interferon-free regimes

The addition of DAAs to PEG-IFN plus RBV as part of triple therapy vastly improved outcomes for patients with CHC. Superior rates of SVR were reported, alongside shortened treatment durations for certain cohorts. However, triple therapy still involved interferon as a mainstay of treatment, bringing with it unpleasant side effects and weekly injections. These factors led to the development of new interferon-free regimens, combining various DAAs, with or without RBV. Interferon-free regimens vary depending on genotype and presence of cirrhosis, and are summarized in Table 2. Those interferon-free regimes currently licensed by the US Food and Drug Administration (FDA) for use in the US are described in the review by Zhang et al.^84^

#### SOF and LDV

The combination of NS5B inhibitor SOF plus NS5A inhibitor LDV (SOF/LDV) is recommended for use in GT1 and GT4–6 patients.^22^ SOF/LDV therapy has been shown to be highly effective in treating GT1 patients, with phase III trials reporting rates of SVR between 94% and 99% in treatment-naïve and treatment-experienced GT1 patients treated with or without RBV for 12 weeks.^85^–^87^ Findings of the ION-3 phase III trial suggested that RBV-free SOF/LDV treatment could be shortened to 8 weeks, but future real-life work is needed to confirm these findings, as no patients with cirrhosis were included in the study.^87^ Post hoc analysis suggested that a viral load of <6,000,000 IU/mL should be the cut-off value when 8-week therapy is used.^88^ However, because HCV RNA level determination may be inaccurate at these values, there is still uncertainty as to whether patients with viral loads of <6,000,000 IU/mL should receive 8- or 12-week SOF/LDV.^89^ Current guidelines recommend the addition of RBV, or extending treatment duration to 24 weeks (without RBV), for patients with negative predictors of response, such as cirrhosis, or those who have failed previous treatment.^22^

Trial data have shown SOF/LDV without RBV to be effective in the treatment of GT4 infection, with 95% (20/21) of patients achieving SVR.^90^ This regimen has also been shown effective in GT6 patients, with 96% (24/25) achieving SVR.^91^ Most recently, a phase II trial involving GT5 patients reported SVR rates of 95% (39/41) in those receiving SOF/LDV without RBV.^92^

#### Ritonavir-boosted PTV and OBV with or without DVR

This combination of DAAs has been shown to be effective in treating GT1 and GT4 infection. PTV, OBV, and DVR are DAAs targeting the NS3/4A, NS5A, and NS5B HCV proteases, respectively. Ritonavir is an inhibitor of the cytochrome (CYP) P450 enzyme CYP3A4, and acts as a pharmacological enhancer of PTV, allowing for once-daily dosing.^93^ Phase III trials have demonstrated the effectiveness of ritonavir-boosted PTV/OBV/DVR in the treatment of GT1 infection. Amongst treatment-naïve patients, SVR rates between 90% and 99% were reported, depending on genotype subtype and addition of RBV to treatment regime.^81^,^82^ SVR rates between 96% and 100% have been reported in phase III trials involving treatment-experienced patients.^83^,^94^ Finally, the TURQUOISE phase III trial reported rates of SVR of 92% (191/208) and 96% (165/172) in patients with cirrhosis receiving 12- and 24-week treatment with PTV/OBV/DVR plus RBV, respectively.^95^

The use of a 12-week regimen of ritonavir-boosted PTV/OBV with RBV (without DVR) in treating GT4 infection is studied in the PEARL-1, AGATE-1, and AGATE-2 trials.^96^–^98^ In the PEARL-1 trial, a total of 91 treatment-naïve and treatment-experienced patients without cirrhosis received this treatment regimen. All 91 achieved SVR12.^96^ The AGATE-1 and AGATE-2 trials added to findings of the PEARL-1 study by including patients with cirrhosis. All study participants in the AGATE-1 trial had cirrhosis, where SVR rates of 97% (59/61) were reported.^97^ The AGATE-2 trial investigated patients with and without cirrhosis. SVR rates of 97% (30/31) and 94% (94/100) were achieved in these cohorts, respectively. Extending treatment duration to 24 weeks did not increase rates of SVR in a sub-cohort of patients with cirrhosis.^98^

#### SOF and SMV

The RBV-free combination of NS5B inhibitor SOF and NS3/4A inhibitor SMV has been shown to be effective in treating GT1 and GT4 infection. The OPTIMIST-1^99^ and OPTIMIST-2^100^ phase III trials investigated outcomes of GT1-infected patients without and with cirrhosis, respectively. Rates of SVR were 97% (150/155) in patients without cirrhosis receiving 12-week treatment with SOF/SMV.^99^ SVR rates were 83% (86/103) in patients with cirrhosis receiving this treatment regimen.^100^ In keeping with existing literature,^58^ the presence of the Q80K polymorphism amongst GT1a patients was associated with lower rates of SVR.^100^

#### SOF and DCV

The use of SOF/DCV with or without RBV has been shown to be effective in treating GT1 and GT2 infection. In a phase II trial, SVR was achieved in 98% (164/167) and 92% (24/26) of GT1 and GT2 patients, respectively.^101^ This combination has also been shown effective in treating GT3 infection, with an SVR rate of 89% reported in a phase II (16/18)^101^ and phase III (135/152) trial.^102^

#### SOF and VEL

The once-daily, RBV-free combination of SOF/VEL has been shown to be an effective pangenotypic therapeutic option. The ASTRAL-1^103^ phase III trial investigated outcomes of patients with GT1, GT2, or GT4–6 infection. GT1 patients achieved SVR rates of 98% (323/328), including 98% (206/210) and 99% (117/118) in patients with GT1a or 1b infection, respectively. SVR rates of 100% (104/104), 100% (116/116), 97% (34/35), and 100% (41/41) were reported in patients with GT2, GT4, GT5, and GT6 infection, respectively.^103^ The ASTRAL-2 and ASTRAL-3 phase III trials investigated outcomes of GT2 and GT3 patients. Confirming findings of the ASTRAL-1 phase III trial, 99% (133/134) of GT2 patients achieved SVR.^103^,^104^ GT3 SVR rates were 95% (264/277), but patients with NS5A resistance-associated variants achieved lower rates of SVR.^104^

#### GZR and EBR

The RBV-free combination of GRZ/EBR has been shown to be effective in treating GT1, GT4, and GT6. The C-WORTHY phase II trial investigated outcomes of GT1 patients. Following a 12-week regimen with GRZ/EBR, SVR rates of 92% (48/52) and 95% (21/22) were reported in GT1a and GT1b patients, respectively.^105^ These findings were confirmed in the subsequent C-EDGE phase III trial, where 92% (144/157) of GT1a and 99% (129/131) of GT1b patients achieved SVR.^106^ The presence of certain NS5A resistance-associated variants was associated with lower responsiveness to therapy.^106^ There are limited data for outcomes of non-GT1 patients. The C-EDGE phase III trial also included GT4 and GT6 patients. SVR was achieved in 100% (18/18) and 80% (8/10) of patients, respectively.^106^ Ideally, further work involving larger patient cohorts is needed to confirm the efficacy of GZR/EBR for GT4/GT6 infection.

## Looking ahead: pangenotypic therapies

The past 25 years have seen a revolutionary change in the treatment of CHC. The discovery of DAAs vastly improved cure rates, with certain combinations curing HCV infection in almost 100% of cases. Once-daily, oral combinations have superseded interferon-based regimens, which were associated with complex dosing schedules, weekly injections, and unpleasant side effects. Treatment duration has also been shortened considerably, reducing side-effect profiles and making treatment regimes more bearable. Ultimately, what was once an incurable disease is now potentially curable in almost all those who are able to access the new standards of care.

In June 2016, the first fixed-dose combination pangenotypic regimen of SOF/VEL was approved by the FDA,^107^ heralding a new era of DAA therapy with an almost “one-size-fits-all”-type management and potentially simplifying management by obviating the need to determine genotype prior to treatment. The ASTRAL-1–5 studies have confirmed the pangenotypic efficacy of SOF/VEL, as well as its effi-cacy in HIV/HCV coinfection and decompensated liver disease.^103^,^104^,^108^,^109^ There is also the desire to reduce treatment durations even further, and it will likely be investigated in future work.

An important factor influencing DAA therapy is drug–drug interactions (especially with antiretrovirals) that alter DAA efficacy through pharmacokinetic interactions as a result of enzyme induction or inhibition of the CYP P450 enzyme subunits involved in the metabolism of DAAs. All potential drug–drug interactions must be checked before initiation of DAA therapy (http://www.hep-druginteractions.org).

One potential alternative method of pangenotypic therapy involves host-targeted agents (HTAs). Rather than targeting the virus directly like DAAs, HTAs act on the host, interfering with cellular factors involved in viral replication.^110^ One such target for HTAs is the hepatic microRNA-122 (miRNA-122), which binds to the HCV genome and enhances viral replication.^111^ The modified oligonucleotide, Miravirsen, sequesters and inhibits miRNA-122, and has been shown to reduce HCV RNA levels in a human phase II trial.^112^ More recently, another miRNA-122 inhibitor, RG-101, has been shown as a potentially valuable addition to HCV treatment. By adding RG-101 to DAA-based therapy, treatment duration was shortened to 4 weeks. At interim analysis, 97% (37/38) of patients achieved SVR at 8 weeks after completing treatment.^113^

By targeting host factors with low genetic variability, HTAs offer a high genetic barrier to resistance.^110^ This is in contrast to DAAs, where resistance may emerge due to the high levels of viral genetic heterogeneity. Consequently, it is thought that the combination of HTAs with DAAs may prevent the emergence resistance, potentially allowing for even shorter treatment periods.^114^ Initial experiences with HTAs such as RG-101 are encouraging,^113^ but we await the results of phase III trials to confirm these findings.

## Potential barriers to HCV eradication, and possible solutions

With the emergence of therapies able to cure HCV in almost all instances, the World Health Organization (WHO) has prioritized global elimination by 2030 as part of their sustainable development goals.^115^ However, in order to achieve this, there are a number of barriers to eradication that must be overcome.

### Therapeutic barriers

One such barrier is the prohibitive cost of DAAs. The latest generation of DAAs is expensive, and there is significant variation in DAA costs between countries.^116^ For example, a 12-week course of SOF costs around £35,000 in the UK, and $84,000 (roughly £55,000 GBP) in the US.^117^ With many countries being limited by finite financial health care resources, these latest, highly efficacious treatments will not be available to everyone. A mechanism of approaching this problem is through treatment prioritization: those who will benefit the most are treated first. However, while treatment prioritization may be a means to manage finite health care resources, it should be accepted that all with CHC warrant therapy and should be treated, except those with an obvious reason not to do so, such as terminal end-stage disease.^22^ One such criterion for treatment prioritization is the degree of fibrosis, including presence of cirrhosis. While liver biopsy and transient elastography are commonly used to assess the degree of liver fibrosis, these tests are expensive and require specialist health care settings and equipment, limiting their use in LMICs. Portable transient elastography offers a cheaper alternative to its fixed counterpart, but with devices costing US$30,000 plus annual maintenance costs of US$4,700, the use of portable transient elastography may not be feasible in many LMICs.^118^ Noninvasive serum-based tests like the aminotransferase/platelet ratio index (APRI) and FIB-4 measure indirect markers of fibrosis such as alanine transaminase, aspartate transaminase, and platelets. The WHO, in its HCV guidelines, recommends the use of such scoring systems in resource-limited settings as an amenable method of disease stratification.^118^

Generic licensing offers an attractive means to upscale therapy. This allows for generic drugs to be manufactured, which are equivalent to “brand drugs” in dosage, strength, route of administration, quality, performance, and intended use.^119^ The advantage of using generic drugs is that they are much cheaper than the originator products. This is particularly advantageous for less economically developed countries where there is less money available for expensive treatments. Generic SOF, SOF/LDV, and DCV are being manufactured under license to the originator companies by pharmaceutical companies in India. Gilead have licensed 11 generic manufacturers in India to allow distribution to 101 countries globally.^120^ In April 2015, the WHO included DCV in its essential medicine list, and in November 2015, Bristol-Myers Squibb allowed the Medicines Patent Pool to pronounce a license and technology transfer agreement for DCV in 112 LMICs allowing for manufacturing of generic DCV globally.^121^

One final way of addressing the issue of expensive treatment is through government subsidization. The Japanese Government heavily subsidized the cost of SOF for patients treated under the national health plan. This means that for every patient treated with a 12-week course of SOF, the government will pay US$43,000, and the patient US$335.^122^ Following the mass anti-schistosomal treatment regime that left millions chronically infected with HCV,^10^ the Egyptian government is now also subsidizing treatment.^123^ Egypt has gone a step further and is producing its own generic SOF and DCV. Similar governmental subsidization schemes are also offered in Australia.^124^

### Diagnostic barriers

A significant challenge facing HCV eradication is that many patients are unaware of their infection. If patients do not know they are infected, then treatment, no matter how effective, will not be administered. This is particularly problematic in LMICs, where the vast proportion of CHC cases are found.^5^ Furthermore, it is imperative to have an idea of the scale of HCV infection before implementing a successful global health intervention. Screening is recommended in at-risk populations, and is based on the detection of anti-HCV antibodies using enzyme immunoassays.^22^ HCV RNA detection is then carried out using PCR to identify viremic patients and monitor treatment progress.^22^ However, such screening tools may not be applicable in LMICs, as they require laboratories with sophisticated equipment and trained staff. When present, such laboratories are often few in number and centralized, limiting access to screening. In addition, even where screening is readily available, the associated financial costs borne by the patient are much higher than in Europe or North America.^125^ Finally, transportation of blood samples to centralized laboratories may be associated with logistical issues, meaning that patients must travel to a clinic nearby in order to be screened.^125^

One alternative to the expensive and complex PCR-based testing involves HCV core-antigen detection. HCV core-antigen levels correlate with HCV RNA levels, thus acting as a surrogate marker of HCV replication.^126^ While highly accurate at diagnosing HCV infection, core-antigen detection is not suitable for determining response-guided therapies, owing to its relative insensitivity at low viral loads.^127^ Nevertheless, LMICs may benefit from the incorporation of core-antigen testing for HCV screening and disease monitoring.

The recent development of rapid diagnostic tests (RDTs) offers an attractive solution to the problems associated with blood collection and transport. RDTs test for infection using blood serum or plasma, or oral fluid.^128^ The major advantage is that they are simple, offer rapid results at room temperature, and require very little training to use.^128^ One such example is the use of dried blood spot (DBS) samples. These involve dried spots of capillary samples collected on filter paper. DBS testing has been shown highly sensitive in detecting anti-HCV antibodies, but relatively insensitive at measuring HCV RNA and core-antigen levels.^128^–^130^ Although genotyping is not always possible with DBS testing,^129^ this is unlikely to matter, as the latest SOF-based regimens offer pangenotype coverage. RDT using oral fluid samples as an alternative to DBS has also been shown effective at detecting anti-HCV antibodies.^130^

## Conclusion

Treatment for CHC has advanced significantly in the last few years. What was once a lifelong condition requiring complex, relatively ineffective treatment regimens with unpleasant side effects can now be cured in almost all instances with a short course of once-daily, all-oral medication, with an SVR associated with an improvement in all-cause and liver-related mortality.^23^–^25^ Thus, all HCV-infected individuals are candidates for treatment, but in resource-constrained countries with a backlog of untreated, HCV-infected individuals, treatment will need to be prioritized. In high-risk groups at risk of reinfection, strategies aimed at behavior modification and harm reduction will be important. While there are still barriers preventing the complete eradication of CHC, shared international efforts to overcome these give cause to be optimistic of what the future of CHC treatment holds.

## Figures and Tables

**Table 1:** Directly acting antivirals and sites of action

Protease inhibitor		
NS3/4A inhibitor	NS5A inhibitor	NS5B inhibitor
Telaprevir	Daclatasvir	Sofosbuvir
Boceprevir	Ledipasvir	Dasabuvir
Simeprevir	Elbasvir	
Grazoprevir	Ombitasvir	
Paritaprevir	Velpatasvir	
Asunaprevir	Odalasvir	
Voxileprevir		
Glecaprevir		

**Table 2:** Interferon-free treatment regimens for chronic hepatitis C according to cirrhosis and treatment status, as recommended by the European Association for the Study of the Liver and AASLD

Genotype	No cirrhosis	
	Treatment naïve	Treatment experienced
1	SOF/LDV (8–12 weeks)	SOF/LDV ± RBV (12 weeks)^a^
	SOF/VEL (12 weeks)	SOF/VEL (12 weeks)
	RTV–PTV/OBV/DSV ± RBV (8-12 weeks)^b^	RTV–PTV/OBV/DSV ± RBV (12 weeks)^b^
	GZR/EBR (12 weeks)^c^	GZR/EBR (12 weeks)^c^
	SOF + DCV (12 weeks)	SOF + DCV ± RBV (12 weeks)^a^
	SOF + SMV (12 weeks)	SOF + SMV (12 weeks)
2	SOF/VEL (12 weeks)	SOF/VEL (12 weeks)
	SOF + DCV (12 weeks)	SOF + DCV (12 weeks)
3	SOF/VEL (12 weeks)	SOF/VEL ± RBV (12 weeks)^d^
	SOF + DCV (12 weeks)	SOF + DCV ± RBV (12 weeks)^d^
4	SOF/LDV (12 weeks)	SOF/LDV ± RBV (12 weeks)^d^
	SOF/VEL (12 weeks)	SOF/VEL (12 weeks)
	RTV–PTV/OBV + RBV (12 weeks)	RTV–PTV/OBV + RBV (12 weeks)
	GZR/EBR (12 weeks)	GZR/EBR (12 weeks)^e^
	SOF + DCV (12 weeks)	SOF + DCV ± RBV (12 weeks)^d^
	SOF + SMV (12 weeks)	SOF + SMV ± RBV (12 weeks)^d^
5/6	SOF/LDV (12 weeks)	SOF/LDV ± RBV (12 weeks)^d^
	SOF/VEL (12 weeks)	SOF/VEL (12 weeks)
	SOF + DCV (12 weeks)	SOF + DCV ± RBV (12 weeks)^d^

**Genotype**	**Cirrhosis**	
	**Compensated: naïve/experienced**	**Decompensated**

1	SOF/LDV ± RBV (12 weeks)^f^	SOF/LDV + RBV (12 weeks)^l^
	SOF/VEL (12 weeks)	SOF/VEL + RBV (12 weeks)^l^
	RTV–PTV/OBV/DSV ± RBV (12/24 weeks)^g^	
	GZR/EBR (12 weeks)^h^	
	SOF + DCV ± RBV (12 weeks)^f^	SOF + DCV + RBV (12 weeks)^l^
	SOF + SMV (12 weeks)	
2	SOF/VEL (12 weeks)	SOF/VEL + RBV (12 weeks)^l^
	SOF + DCV (12 weeks)	SOF + DCV + RBV (12 weeks)^l^
3	SOF/VEL (12 weeks)^i^	SOF/VEL + RBV (24 weeks)^l^
	SOF + DCV + RBV (24 weeks)	SOF + DCV + RBV (24 weeks)^l^
4	SOF/LDV ± RBV (12 weeks)^j^	SOF/LDV + RBV (12 weeks)^l^
	SOF/VEL (12 weeks)	SOF/VEL +RBV (12 weeks)^l^
	RTV–PTV/OBV + RBV (12 weeks)	
	GZR/EBR (12 weeks)^k^	
	SOF + DCV ± RBV (12 weeks)^j^	SOF + DCV + RBV (12 weeks)
	SOF + SMV ± RBV (12 weeks)^j^	
5/6	SOF/LDV ± RBV (12 weeks)^j^	SOF/LDV + RBV (12 weeks)^l^
	SOF/VEL (12 weeks)	SOF/VEL + RBV (12 weeks)^l^
	SOF + DCV ± RBV (12 weeks)^j^	SOF + DCV + RBV (12 weeks)^l^

**Notes:**

a If GT1a, add RBV or extend to 24 weeks without RBV.

b If GT1a, add RBV and treat for 12 weeks.

c If GT1a with viral load >800,000 IU/mL, extend to 16 weeks plus RBV.

d Treat for 12 weeks with RBV or 24 weeks without RBV.

e If viral load >800,000 IU/mL, extend to 16 weeks plus RBV.

f If GT1a treatment-experienced, give 12 weeks with RBV or 24 weeks without.

g If GT1a, 24 weeks with RBV. If GT1b, 12 weeks without RBV.

h If GT1a with viral load >800,000 IU/mL, extend to 16 weeks plus RBV.

i If resistance-associated mutations, give 12 weeks with RBV or 24 weeks without.

j If treatment-experienced, give 12 weeks with RBV or 24 weeks without.

k If treatment-experienced with viral load >800,000 IU/mL, extend to 16 weeks plus RBV.

l If intolerant to RBV, give 24-week therapy without RBV.

**Abbreviations:** AASLD, American Association for the Study of Liver Diseases; SOF, sofosbuvir; LDV, ledipasvir; RBV, ribavirin; VEL, velpatasvir; RTV, ritonavir; PTV, paritaprevir; OBV, ombitasvir; DSV, dasabuvir; GZR, grazoprevir; EBR, elbasvir; DCV, daclatasvir; SMV, simeprevir; GT, genotype.
